# Six Novel Biomarkers for Diagnosis and Prognosis of Esophageal squamous cell carcinoma: validated by scRNA-seq and qPCR

**DOI:** 10.7150/jca.50443

**Published:** 2021-01-01

**Authors:** Liuhai Zheng, Linzhi Li, Jun Xie, Hai Jin, Naishuo Zhu

**Affiliations:** 1 Laboratory of Molecular Immunology, State Key Laboratory of Genetic Engineering, School of Life Sciences, Fudan University, Shanghai, China.; 2 Department of Thoracic Surgery, Changhai Hospital, Second Military Medical University, Shanghai, China.

**Keywords:** ESCC, biomarkers, diagnostic, poor prognosis, therapeutic targets

## Abstract

Esophageal squamous cell carcinoma (ESCC) is one of the most common cancers worldwide. ESCC has a generally poor prognosis and there is a lack of available biomarkers for diagnosis and prognosis. The aim of the study was to identify novel biomarkers for ESCC. We screened the overlapping differentially expressed genes (DEGs) acquired from six Gene Expression Omnibus (GEO) ESCC datasets and The Cancer Genome Atlas (TCGA) ESCC datasets. Subsequently, protein-protein interaction network analysis was performed to identify the key hub genes. Then, Kaplan Meier survival and receiver operating curve (ROC) analysis were utilized to clarify the diagnostic and prognostic role of these hub genes. The UALCAN database, single cell RNA sequencing (scRNA-seq) and real-time quantitative PCR (qPCR) were performed to confirm the expression levels of identified hub genes. Finally, immune infiltration analysis was conducted to investigate the role of these genes in the pathogenesis of ESCC. The results showed that PBK, KIF2C, NUF2, KIF20A, RAD51AP1, and DEPDC1 effectively distinguish ESCC tissues from normal samples, and all of them were significantly correlated with overall survival. The results of scRNA-seq and qPCR indicated that the expression levels of hub genes in ESCC were significantly higher than in normal cells or tissues. Further immune infiltration analysis showed that infiltration of dendritic cells was significantly negatively correlated with PBK, KIF2C, NUF2, RAD51AP1, and DEPDC1 expression levels. In conclusion, our results suggest that PBK, KIF2C, NUF2, KIF20A, RAD51AP1 and DEPDC1 are all potential biomarkers for ESCC diagnosis and prognosis may also be potential therapeutic targets for ESCC.

## Introduction

Esophageal cancer was the seventh most common cancer and the sixth leading cause of cancer death worldwide in 2018, with around 572,000 new cases and 509,000 deaths annually 1. As the predominant subtype of esophageal cancer, esophageal squamous cell carcinoma (ESCC) comprises over 90% of all esophageal cancer cases in parts of Asia 2. Many patients with ESCC are diagnosed at an advanced stage due to the lack of effective biomarkers, and often at this stage distant metastases have already occurred resulting in poor prognosis. At present, there is no effective treatment strategy together with the lack of effective diagnosis and prognosis biomarkers, the 5-year survival rate of ESCC patients is less than 30% 2. Moreover, ESCC patients at the advanced stage are always suffering great pains, such as difficulty eating and breathing, which are usually refractory to treatment. Therefore, there is an urgent need to identify more effective biomarkers for ESCC, which will increase the efficiency of diagnosis and treatment, and even improve our understanding of the pathogenesis mechanisms.

In recent years, as high-throughput microarray platforms have been widely used in medical research, a large number of high-throughput data available in many databases, and re-analysis of these data has become an effective and low-cost method to discover biomarkers for many diseases. The Cancer Genome Atlas (TCGA) is the largest database for storing cancer related high-throughput data, including 33 cancer types with more than 20,000 primary cancers and matched normal samples. The Gene Expression Omnibus (GEO) database is a comprehensive repository of high-throughput experimental data in the National Center for Biotechnology Information (NCBI) which is one of the world's largest database of biochips. To date, the GEO database contains > 40000 microarray gene expression datasets and > 2000 items containing the keywords “esophageal cancer”. Currently, these two databases are widely used for data mining due to their large sample size and complete clinical information. Many studies have identified various biomarkers for ESCC based on TCGA and GEO databases 3-9. For example, Mao et al. identified a seven-lncRNA signature to predict overall survival in ESCC, which displayed better prognostic predict ability than tumor-node-metastasis (TNM) stage. Song et al. identified PDLIM2 as a novel prognostic predictor for ESCC, which also associated with nodal invasion. These studies have promoted our understanding of the development of new diagnostic and prognostic biomarkers for ESCC. However, biomarkers related to ESCC diagnosis and prognosis was usually identified independently, which may hinder our understanding of the process from initiation to deterioration. More importantly, most previous studies were mainly depended on one or two datasets, while individual dataset often can be unreliable due to bias introduced by sample processing and insufficient samples 10. Therefore, to obtain more convincing results, more datasets from multiple platforms are needed. What's more, most previous studies based on TCGA or GEO data analysis mainly focused on bioinformatics analysis, and few studies further verified on clinical samples. As we all know, there are certain technical errors in both microarray and next-generation sequencing data. Therefore, to eliminate technical errors, experimental confirmation is required. Finally, as the traditional bulk profiles represents the average expression levels of the constituent cells (malignant, immune and stromal cells), it does not reflect the true condition of cancer cells. Thus, verifying the identified differential expression genes (DEGs) at the single cell resolution will increase the authenticity and reliability of the analysis results, and even improve our understanding of the underlying mechanism. As far as we know, there is no relevant research to verify the identified biomarkers at the single cell levels.

In the present study, we combined six GEO microarray datasets to screen the DEGs in at least two different ESCC datasets, and further integrated with TCGA ESCC dataset to screen the possible biomarkers associated with diagnosis and prognosis for ESCC. We discovered that PBK, KIF2C, NUF2, KIF20A, RAD51AP1, DEPDC1 are possible diagnostic and prognostic biomarkers for ESCC. Then, UALCAN database, scRNA-seq and qPCR were conducted to validate the expression levels of identified genes. Furthermore, we performed immune infiltration analysis to gain a better understanding of the function of these genes. Our study could provide novel biomarkers for ESCC diagnosis and prognosis, and potential targets for ESCC therapy.

## Materials and Methods

### Data collection

**Figure 1:**
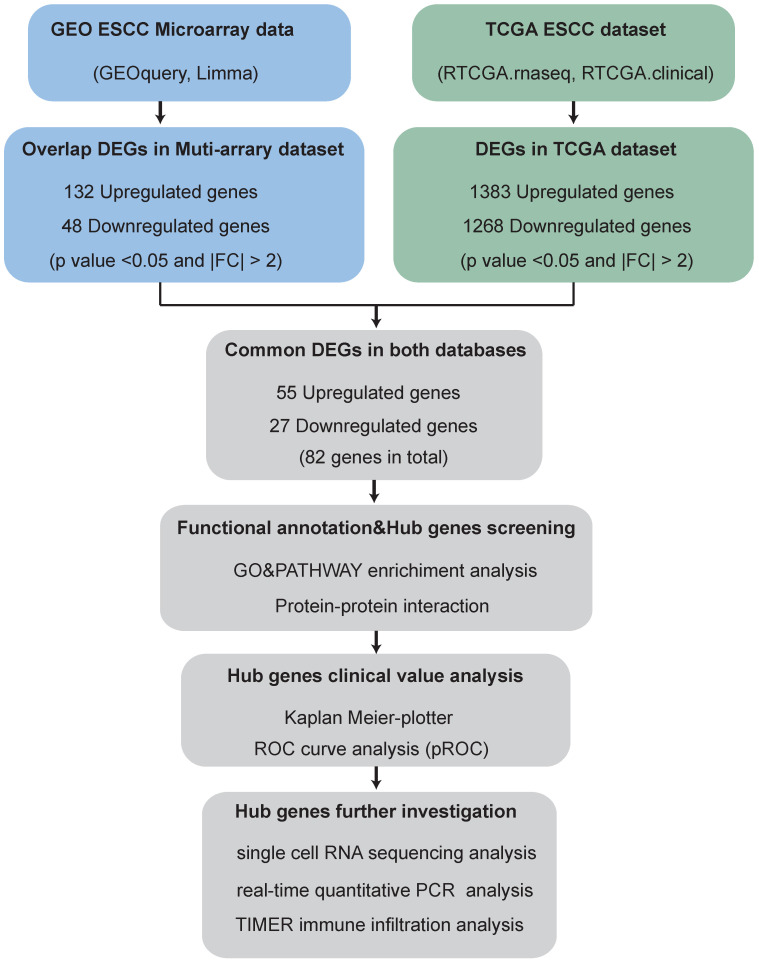
Flowchart for screening novel biomarkers in ESCC.

The brief flowchart for screening novel biomarkers for ESCC is showed in **Fig. 1.**

ESCC-related GEO datasets with primary tumor tissues and matched normal tissues, principal component analysis (PCA) analysis can well distinguish tumor samples from normal samples, expression profiling by array were enrolled into the present study. Thus, the datasets for ESCC (GSE17351, GSE20347, GSE23400, GSE100942, GSE38129 and GSE77861) were downloaded from the GEO (https://www.ncbi.nlm.nih.gov/geo/) database by R ×64 3.6.0 with the “GEOquery” R package 11. The details of these datasets are listed in **Table 1.** TCGA-ESCC samples with RNA-seq data and matched clinical metadata (including 11 normal and 78 tumor samples) were downloaded from the GDC database (https://portal.gdc.cancer.gov/). The detailed clinical characteristics of the enrolled patients in TCGA are shown in **Table 2.**

### Ethics approval and consent to participate

The ethics committee at the changhai hospital, Second Military Medical University approved this study, and written informed consent on the use of clinical specimens from all participants. Six patients with ESCC who underwent surgical resection of tumor tissues without any treatment were enrolled for qPCR assay. Paired adjacent nontumor tissues from the proximal resection margins (>5 cm away from the ESCC sample) were also collected for RNA extraction and qPCR assay. The detailed clinic parameters of these participants are listed in **Table 3.**

### Overlapping DEG analysis

The GEO datasets (GSE17351, GSE20347, GSE23400, GSE100942, GSE38129 and GSE77861) and TCGA datasets were processed using the “Limma” R package 12. A *P*‐value < 0.05 and |FC| > 2 were used as the threshold to identify DEGs. Next, we used an online tool, jvenn (http://jvenn.toulouse.inra.fr/app/index.html) to find overlapping DEGs in at least two of the GEO datasets 13. Finally, the overlapping DEGs were analyzed to identify the most commonly deregulated genes across datasets.

### Functional annotation and hub genes screening

STRING (https://string‐db.org/) was used for Gene ontology (GO), pathway enrichment and protein‑protein interaction (PPI) analysis 14. The PPI information was downloaded and imputed into Cytoscape software (Cytoscape_v3.7.2) to construct a PPI network. In addition, cytoHubba (integrated into Cytoscape software) was applied to screen hub genes using the Maximal Clique Centrality (MCC) method 15.

### Hub gene clinical value analysis

To evaluate the prognostic significance of identified hub genes, a Kaplan Meier survival analysis was carried out using Kaplan Meier-plotter (https://kmplot.com/analysis/) 16. A receiver operating curve (ROC) analysis was conducted using the “pROC” R package to explore the diagnostic value of these hub genes 17.

### Hub genes mRNA expression validation

UALCAN (http://ualcan.path.uab.edu/), which contains TCGA and MET500 transcriptome sequencing data, was used to validate mRNA expression of the hub genes 18.

The scRNA-seq data (SRP119465) which contains three ESCC patients and 208 single cells were downloaded from the Sequence Read Archive (https://www.ncbi.nlm.nih.gov/sra) 19. The detailed clinical characteristics are shown in **Table 4.** Subsequently, Trimmomatic tool were used to remove low quality and adapter reads, and then mapped to the human genome GRCh38 transcriptome using Bowtie. Cell cluster analysis was performed using the “Seurat” R package. Based on the clustering of cell subsets and characteristic gene expression, we annotated the cell subsets and displayed the expression levels of hub genes in all cell subsets.

To detect the mRNA expression of hub genes, qPCR was conducted on the LightCycler® 480 II real-time PCR system (Roche Molecular Diagnostics Inc., South Branchburg, NJ, USA). Briefly, total RNA was extracted from 6 ESCC tissues and adjacent normal tissues using the Trizol reagent (Thermo Fisher Scientific, USA). Single strand complementary DNA (cDNA) was synthesized from 0.5 μg of total RNA using the transcriptor first strand cDNA synthesis kit (Roche Molecular Diagnostics Inc., South Branchburg, NJ, USA). qPCR was performed to quantify the hub genes mRNA expression level using the LightCycler® 480 SYBR Green I Master kit (Roche Molecular Diagnostics Inc., South Branchburg, NJ, USA). The primers used are listed in **Table 5.** Then, the following cycling conditions were applied: 95°C for 5 minutes, followed by 40 cycles of 95°C for 10 seconds and 62°C for 30 seconds. GAPDH served as an internal control to normalize the expression. The 2^-ΔΔCt^ method was employed to calculate the relative expression level.

### Immune infiltration analysis

The correlation of hub gene expression with immune infiltration level was performed using the Tumor Immune Estimation Resource (TIMER, https://cistrome.shinyapps.io/timer/) 20. The TIMER database incorporates 32 cancer types and six immune cell types (B cell, CD4 T cell, CD8 T cell, neutrophil, macrophage, and dendritic cell).

## Results

### Data preprocessing

**Figure 2:**
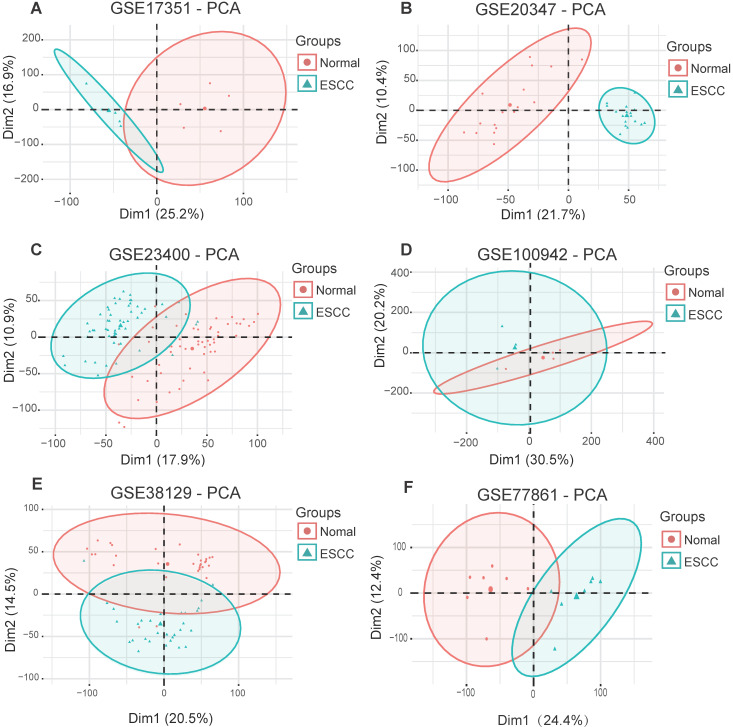
Principal component analysis for ESCC datasets from the GEO database. **(A)** GSE17351. **(B)** GSE20347.** (C)** GSE234300.** (D)** GSE100942. **(E)** GSE38129.** (F)** GSE77861.

To obtain biological changes in gene expression in ESCC, all data were pre-processed. Firstly, we used the “Limma” R package to standardize the data and eliminate the effects of experimental techniques on gene expression. Next, the “factoMineR” and “factoextra” R package were used for principal component analysis (PCA) for quality control. The results showed that all samples (GSE17351, GSE20347, GSE23400, GSE100942, GSE38129 and GSE77861) were clearly divided into normal and tumor groups, except for dataset GSE23400, where four normal samples (GSM573926, GSM573867, GSM573888 and GSM573889) and three tumor samples (GSM573926, GSM573935 and GSM573944) showed no difference between normal tissue and tumors; therefore, these were removed from further analyses (**Fig. 2A-F**).

### Common DEGs in GEO and TCGA datasets

**Figure 3:**
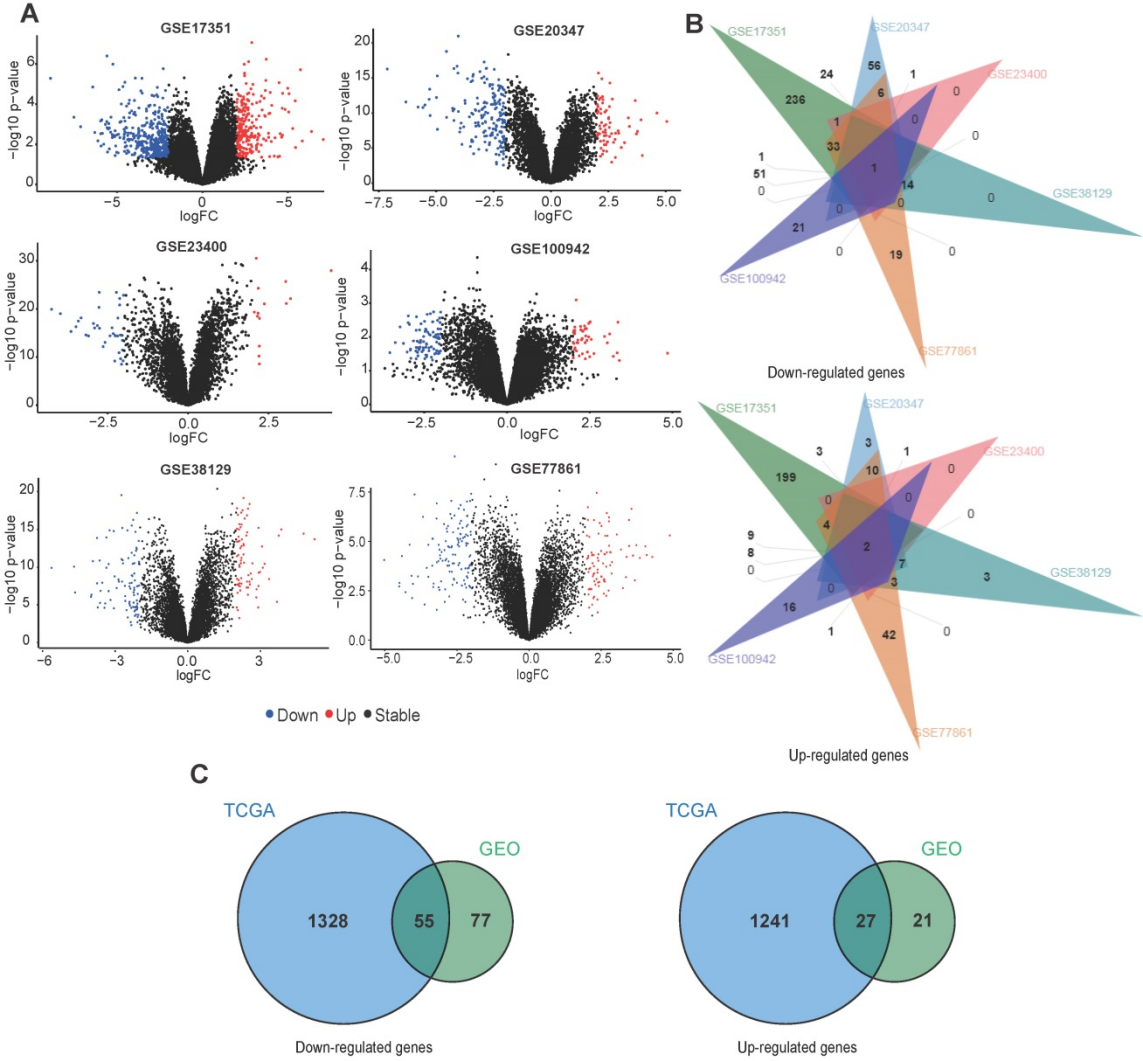
Overlap between DEGs across different ESCC datasets. **(A)** The volcano plots of DEGs obtained from GEO datasets. **(B)** Venn diagram demonstrating the overlap between DEGs in the different GEO datasets.** (C)** Venn diagram displaying the overlap between DEGs from the GEO and TCGA datasets.

For GEO datasets, each set of DEGs was screened separately. The ggplot2 package was used to display the DEGs identified from each dataset (**Fig. 3A**). Table 1 describes details of the DEGs in each dataset. Next, an online tool (jvenn) was used to identify overlapping DEGs in at least two GEO datasets. We found 132 downregulated and 48 upregulated genes (**Fig. 3B**), which were used for identifying common DEGs within the TCGA data. For TCGA datasets, we found 1383 downregulated and 1268 upregulated genes (**Table 1**). **Fig. 3C** shows a venn diagram demonstrating the common DEGs between the GEO and TCGA datasets, 55 downregulated and 27 upregulated genes were found.

### Functional annotation and PPI analysis

**Figure 4:**
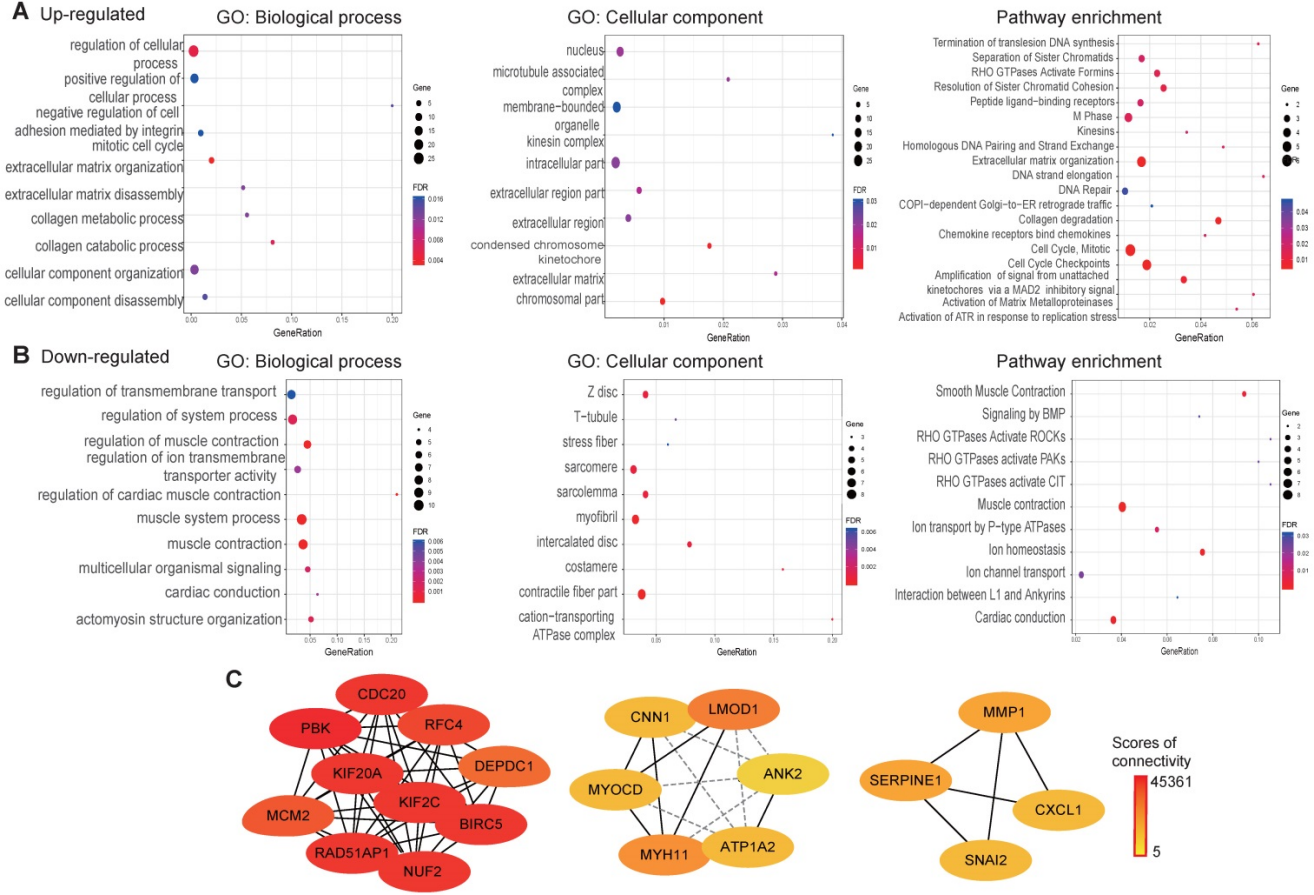
GO, pathways enrichment, and PPI of overlap DEGs in ESCC. **(A)** GO and pathway enrichment results for up-regulated DEGs.** (B)** GO and pathway enrichment results for down‑regulated DEGs. **(C)** PPI network of overlap DEGs from GEO data and TCGA datasets.

To explore the function of the common DEGs, GO, pathway enrichment and PPI analysis were conducted using the STRING database. The results revealed that most of the upregulated genes are found in the nucleus, as membrane-bounded organelles and in the intracellular compartment. The upregulated genes were mainly involved in positive regulation of cellular process, the mitotic cell cycle and collagen metabolic processes. Pathway enrichment analysis revealed that the upregulated genes were mainly involved in the cell cycle extracellular matrix organization and DNA repair (**Fig. 4A**). Cellular component analysis of the downregulated genes showed association with contractile fibers, myofibrils, and intercalated discs. With regard to biological processes, the downregulated genes were associated with muscle system processes, regulation of ion transmembrane transporter activity and actomyosin structure organization. As for pathway enrichment, muscle contraction, ion homeostasis and rho GTPases activation was enriched (**Fig. 4B**). A PPI network was constructed to screen hub genes using the STRING database. Subsequently, the PPI network information was imported into Cytoscape and the MCC method in cytoHubba was applied to screen hub genes. The results showed that PBK, CDC20, KIF2C, BIRC5, NUF2, KIF20A, RAD51AP1, RFC4, MCM2, and DEPDC1 interact with each other with high scores for connectivity (**Fig. 4C**).

### Kaplan Meier survival and ROC analysis

**Figure 5:**
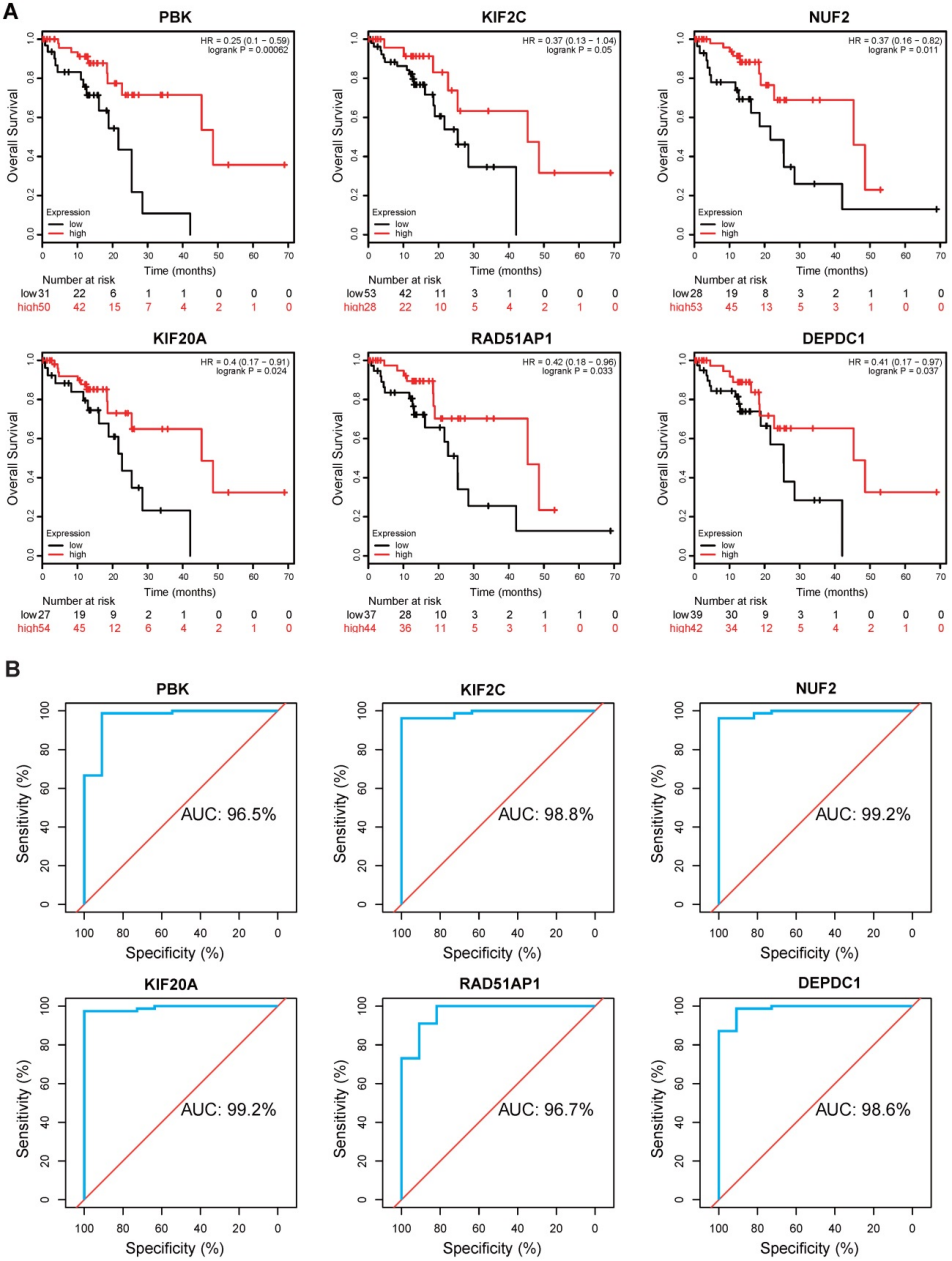
Kaplan-Meier survival and ROC analysis. **(A)** Kaplan Meier survival analysis of PBK, KIF2C, NUF2, KIF20A, RAD51AP1 and DEPDC1 in ESCC. HR: Hazard Ratio.** (B)** ROC analysis of hub genes in ESCC. AUC: area under the curve.

To evaluate the clinical value of these hub genes in ESCC, Kaplan-Meier survival analysis was performed using Kaplan Meier-plotter. Of the 10 genes, we found that PBK (hazard ratio [HR]=0.25, logrank *P=* 0.00062), KIF2C (HR=0.37, logrank *P=* 0.05), NUF2 (HR=0.37, logrank *P=* 0.011), KIF20A (HR=0.4, logrank *P=* 0.024), RAD51AP1 (HR=0.42, logrank *P=* 0.033), and DEPDC1 (HR=0.41, logrank *P=* 0.037) were significantly correlated with overall survival (**Fig. 5A**). ESCC patients with low expression levels of these genes generally have worse survival. Thereafter, a ROC analysis was conducted to investigate diagnostic value. The results showed PBK (area under the curve [AUC]= 96.5%), KIF2C (AUC= 98.8%), NUF2 (AUC= 99.2%), KIF20A (AUC= 99.2%), RAD51AP1 (AUC= 96.7%), and DEPDC1 (AUC= 98.6%) effectively distinguish ESCC tissues from normal samples (**Fig. 5B**).

### Hub gene expression levels validation

**Figure 6:**
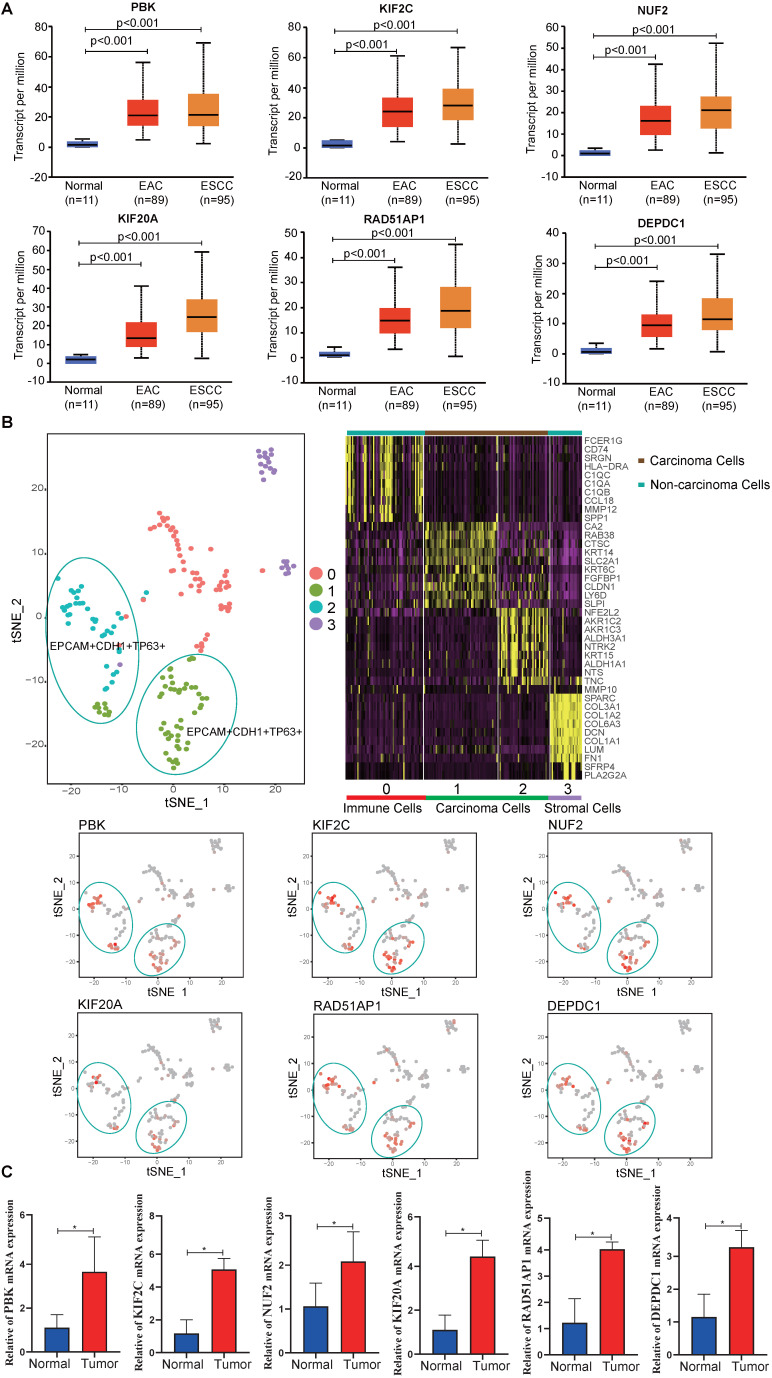
Expression levels of hub genes.** (A)** Expression levels of PBK, KIF2C, NUF2, KIF20A, RAD51AP1 and DEPDC1 in UALCAN database.** (B)** scRNA-seq analysis of hub genes in ESCC. **(C)** The relative mRNA expression of PBK, KIF2C, NUF2, KIF20A, RAD51AP1 and DEPDC1 were confirmed by qPCR (n=6). Data are presented as the means ± SEM. *P < 0.05 represent significant differences between the indicated groups.

To validate the hub gene expression levels, UALCAN ESCA data, which contains 11 normal, 89 esophageal adenocarcinoma (EAC) and 95 ESCC samples, was used for analysis. The results showed that the mRNA expression levels of hub genes (PBK, KIF2C, NUF2, KIF20A, RAD51AP1 and DEPDC1) in tumor samples (EAC, ESCC) were significantly higher than in normal samples (*P <* 0.05) (**Fig. 6A**). In addition, the scRNA-seq analysis showed that the hub genes were mainly expressed on carcinoma cells, indicating that the identified differentially expressed genes were caused by cancer cells (**Fig. 6B**). To eliminate errors caused by sequencing, qPCR was performed. We found that all hub genes mRNA expression levels were significantly elevated in tumor tissues compared to adjacent normal tissues (*P <* 0.05) (**Fig. 6C**).

### Immune infiltration analysis

**Figure 7:**
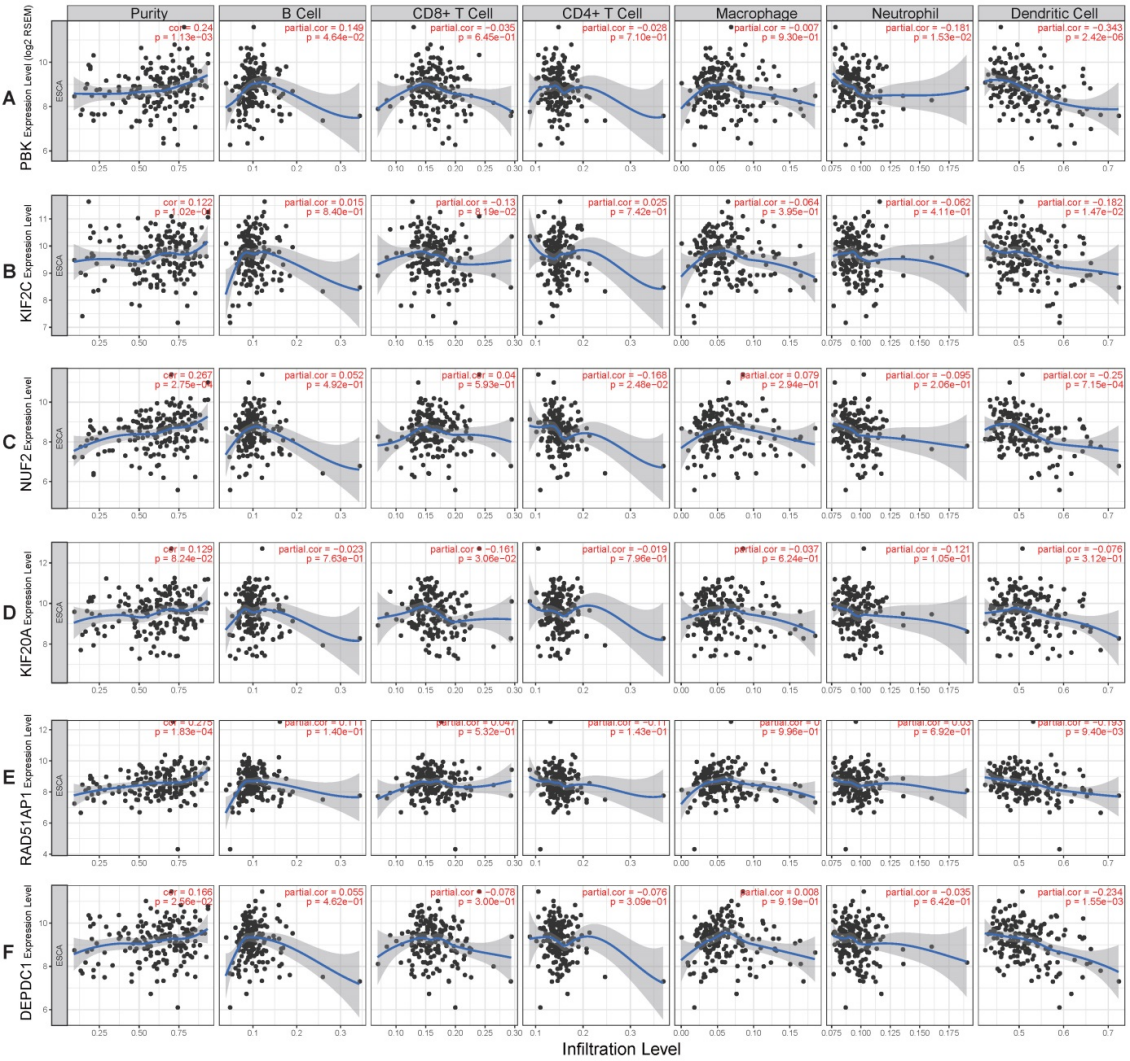
Correlation of hub genes expression levels with immune infiltration of B cell, CD4 T cell, CD8 T cell, neutrophil, macrophage, and dendritic cell in ESCC. **(A)** PBK. **(B)** KIF2C.** (C)** NUF2.** (D)** KIF20A. **(E)** RAD51AP1.** (F)** DEPDC1.

To better understand the function of these genes (PBK, KIF2C, NUF2, KIF20A, RAD51AP1, DEPDC1), the relationship between their expression and immune infiltration was performed. The results indicated that tumor purity significantly and positively correlated with PBK (R= 0.24, *P=* 1.13e-03), NUF2 (R= 0.267, *P=* 2.75e-04), RAD51AP1 (R= 0.275, *P=* 1.83e-04), and DEPDC1 (R= 0.166, *P=* 2.56e-02) expression. Infiltration of dendritic cells was significantly negatively correlated with PBK (R= -0.343, *P=* 2.42e-06), KIF2C (R= -0.182, *P=* 1.47e-02), NUF2 (R= -0.25, *P=* 7.15e-04), RAD51AP1 (R= -0.193, *P=* 9.4e-03), and DEPDC1 (R= -0.234, *P=* 1.55e-03) expression levels. However, their expression had no obvious correlation with infiltration of other immune cells (B cell, CD4 T cell, CD8 T cell, neutrophil, macrophage) (**Fig. 7A-F**).

## Discussion

ESCC is the predominant type of esophageal carcinoma worldwide. In China, ESCC is the fourth leading cause of cancer-related death 21. Despite recent improvements in diagnosis and treatment, ESCC's prognosis is still poor. The 5-year survival rate of ESCC patients is less than 30% 2. Although several biomarkers for ESCC have been identified, the clinical value of most of them has not been confirmed. Thus, screening for more efficient biomarkers for ESCC diagnosis and prognosis is urgently required.

Integrating multiple datasets is considered to be a better method to improve the reliability of the results than individual dataset analysis. In our study, we integrated six GEO microarray datasets with TCGA ESCC dataset to screen the possible biomarkers for ESCC. In addition, in order to improve the reliability of the results, scRNA-seq and qPCR were performed to confirm the expression level of the identified genes. We identified six novel biomarkers (PBK, KIF2C, NUF2, KIF20A, RAD51AP1, DEPDC1) that are related to the diagnosis and prognosis of ESCC.

PBK is a serine-threonine kinase that was reported upregulated in breast cancer, and server as a therapy target for breast cancer 22. Moreover, PBK was also overexpressed in oral cancer, and is known to be a favorable prognostic indicator for oral cancer 23. Ohashi et al found that PBK overexpressed is associated with worse outcomes in ESCC 24. However, our results revealed that high PBK expression significantly correlated with better outcomes (HR=0.25, logrank *P=* 0.00062). The contradictory result may be due to bias in individual cohort studies. The detailed reasons need to be further validated. In addition, ROC analysis also showed that PBK can effectively distinguish ESCC tissues from normal samples (AUC= 96.5%), indicating that PBK could be an independent diagnostic biomarker for ESCC.

KIF2C, a member of the motor proteins family, functions as a microtubule-dependent molecular motor 25. Previous studies have demonstrated that high KIF2C expression can serve as an independent marker of poor prognosis in several tumors, including glioma, colorectal cancer, and gastric cancer 26-28. In contrast, our study showed that low expression levels of KIF2C correlated with worse survival (HR=0.37, logrank *P=* 0.05). The diagnostic role of KIF2C in ESCC has not been previously reported. Our study revealed that KIF2C could be a useful diagnostic biomarker for ESCC (AUC= 98.8%).

NUF2 is a component of a protein complex associated with the centromere that plays an important role in chromosome segregation 29. Previous studies have shown that NUF2 is an effective prognostic molecule for hepatocellular carcinoma 30, and silencing NUF2 can suppress human hepatocellular carcinoma tumor growth and induce apoptosis 31. In addition, NUF2 overexpression is also related to poor prognosis in pancreatic cancer 32. However, the role of NUF2 in ESCC still unknown. Our results revealed that low expression in ESCC is associated with worse outcomes, and NUF2 can efficiently distinguish tumor tissues from normal tissue (AUC= 99.2%), suggesting its diagnostic value for ESCC.

KIF20A (also named RAB6KIFL), has been reported overexpressed in many cancers including pancreatic cancer, melanoma, breast cancer, and glioma 33-37. Moreover, KIF20A was also reported as a prognostic indicator for cervical squamous cell carcinoma, ovarian clear‑cell carcinoma, and glioma 37-39. However, the role of KIF20A in ESCC has never been reported. In the present study, we found that KIF20A is overexpressed in ESCC, and low expression was associated with poor prognosis. ROC analysis revealed KIF20A is a promising diagnostic biomarker for ESCC (AUC= 99.2%).

RAD51AP1 is a DNA-binding protein, which plays a key role in homologous recombination and DNA repair 40. Upregulated RAD51AP1 has been reported to be associated with poor prognosis in ovarian and lung cancers 41, 42. In the present study, we found that RAD51AP1 is an effective diagnostic biomarker for ESCC (AUC= 96.7%). Moreover, silencing of RAD51AP1 can inhibit epithelial-mesenchymal transition and metastasis in non-small cell lung cancer 41, 42. Therefore, high expression of RAD51AP1 may be implicated in ESCC development.

DEPDC1 is a novel cancer-related gene that was reported to be overexpressed in many tumors including bladder cancer, multiple myeloma, breast cancer, colorectal cancer, and hepatocellular carcinoma 43-47, and is known to be a poor prognostic indicator for these tumors. However, its role in ESCC has never been reported. Herein, our results revealed that DEPDC1 is a promising diagnostic and prognostic biomarker for ESCC.

So far, a great number of potential biomarkers have been identified. However, there is still a great gap to put these findings into clinical application. The major reason is that the poor reproducibility, small overlap between studies, and low sensitivity and specificity for diagnosis and prognosis of ESCC. For example, Takeshita et al. found that the sensitivity and specificity of serum miR-1246 for the diagnosis of ESCC were 71.3% and 73.9%, respectively 48. The specificity and sensitivity of miR-146a for diagnosis of ESCC were 68.6% and 85.7% 49. Additionally, Adams et al. speculated that the four indicators of AHRR, p16INK4a, MT1G and CLDN3 can be combined to improve the sensitivity and specificity for the diagnosis of ESCC. However, in clinical applications, the sensitivity and specificity of the combined index in the diagnosis of ESCC were only 50% and 68%, respectively 50. In our present study, we found that PBK (area under the curve [AUC]= 96.5%), KIF2C (AUC= 98.8%), NUF2 (AUC= 99.2%), KIF20A (AUC= 99.2%), RAD51AP1 (AUC= 96.7%), and DEPDC1 (AUC= 98.6%) all with a high sensitivity and specificity for the diagnosis of ESCC. However, future work focusing on the *in vitro* and *in vivo* validation before clinical application is still needed.

## Conclusions

In conclusion, our study identified six novel biomarkers (PBK, KIF2C, NUF2, KIF20A, RAD51AP1 and DEPDC1) for ESCC. Notably, all of them could be independent diagnostic and prognostic indicators for ESCC. In addition, scRNA-seq results showed that the hub genes is mainly expressed on carcinoma cells, and qPCR results also indicated that the expression of the hub genes in tumor tissues is significantly higher than normal tissues. All these results confirmed the reliability of the analysis. Moreover, their expression levels were significantly positively correlated with tumor purity, and negatively correlated with the infiltration of DCs. DCs are the most important antigen presenting cells, which play a key role in connecting innate immunity with acquired immunity. Reducing the infiltration of DCs may affect the presentation of antigens, resulting in the inability of the host immune response to effectively kill tumor cells. This may be one of the underlying molecular mechanisms in the tumorigenesis of ESCC. Our results indicate that these genes may also be potential targets for ESCC therapy. Further experiments are required to confirm these findings.

## Figures and Tables

**Table 1:** Details of ESCC datasets from the GEO and TCGA database

GSE	Publication	Upregulated DEG	Downregulated DEG	Platform	Sample size
GSE17351	Carcinogenesis	266	426	GPL570	Tumor: 5
					Normal: 5
GSE20347	BMC Genomics	69	183	GPL571	Tumor: 17
					Normal: 17
GSE23400	Clin Cancer Res	14	37	GPL96	Tumor: 53
					Normal: 53
GSE100942	Theranostics	46	83	GPL570	Tumor: 4
					Normal: 4
GSE38129	BMC Genomics	74	93	GPL571	Tumor: 30
					Normal: 30
GSE77861	BMC Cancer	88	123	GPL570	Tumor: 7
					Normal: 7
TCGA		1268	1383	IlluminaHiSeq	Tumor: 78
					Normal: 11

**Table 2:** Clinical characteristics of the enrolled patients in TCGA

Characteristics	Number of sample size (%)
**Age (years)**	
<50	15 (18.5)
≥50	66 (81.5)
**Gender**	
Female	12 (14.8)
Male	69 (85.2)
**Stage**	
IA	3 (3.7)
IB	4 (4.9)
IIA	35 (43.2)
IIB	13 (16.1)
III	9 (11.1)
IIIA	8 (9.9)
IIIB	3 (3.7)
IV	5 (6.2)
NA	1 (1.2)
**T classification**	
T1	8 (9.9)
T2	28 (34.6)
T3	40 (49.4)
T4	5 (6.1)
**N classification**	
N0	46 (56.8)
N1	25 (30.9)
N2	5 (6.2)
N3	1 (1.2)
Nx	4 (4.9)
**M classification**	
M0	71 (87.6)
M1	5 (6.2)
Mx	5 (6.2)
**Radiotherapy**	
Yes	37 (45.7)
No	44 (54.3)
**Chemotherapy**	
Yes	29 (35.8)
No	52 (64.2)
**Vital status**	
Dead	16 (19.8)
Alive	65 (80.2)

Abbreviation: NA, not available.

**Table 3:** Clinic parameters of enrolled patients in the current study for qPCR assay

	ESCC01	ESCC02	ESCC03	ESCC04	ESCC05	ESCC06
TNM	T3N0Mx	T3N0Mx	T3N0Mx	T3N0Mx	T3N1Mx	T3N3Mx
Stage	IIA	IIA	IIA	IIA	IIIB	IIIC
Gender	Female	Male	Male	Male	Male	Male
Age	66	70	61	56	64	71
Sampling Method	Surgical	Surgical	Surgical	Surgical	Surgical	Surgical
Sampling Time	2018.05.31	2018.08.24	2019.01.03	2019.06.27	2019.07.09	2019.07.11
Treatment	Surgery	Surgery	Surgery	Surgery	Surgery	Surgery
Vital Status	Alive	Alive	Alive	Alive	Alive	Alive

**Table 4:** Clinic parameters of enrolled patients in scRNA-seq dataset

	ESCC01	ESCC02	ESCC03
TNM	T3N0M0	T3N1M0	T3N1M0
Stage	IIA	IIIB	IIIB
Gender	Male	Male	Female
Sampling Method	Biopsy	Biopsy	Surgical
Sampling Time	2015.10.28	2015.10.22	2015.07.30
Treatment	Radiotherapy	Radiotherapy	Surgery
Vital Status	Alive	Alive	Alive

**Table 5:** Prime sequences

Target	Accession	Orientation	Primers sequence (5′-3′)
gene	number		
GAPDH	NM_001256799	Forward	GGAGCGAGATCCCTCCAAAAT
		Reverse	GGCTGTTGTCATACTTCTCATGG
PBK	NM_018492	Forward	TAGGAGTCTCTCTACCACTGGA
		Reverse	TCCCACAAAGTAAGGCCAAAG
KIF2C	NM_006845	Forward	CTCAGTTCGGAGGAAATCATGTC
		Reverse	TGCTCTTCGATAGGATCAGTCA
NUF2	NM_031423	Forward	TGTTAAGCAATACAAACGCACAG
		Reverse	TGCCTTTTCAATACCGTCGTG
KIF20A	NM_005733	Forward	TTGAGGGTTAGGCCCTTGTTA
		Reverse	GTCCTTGGGTGCTTGTAGAAC
RAD51AP1	NM_001130862	Forward	TGGTGGTGTTCAAGGGAAAAG
		Reverse	AGGTGCAAAGTCTGGTTCAGT
DEPDC1	NM_001114120	Forward	ATGCGTATGATTTCCCGAATGAG
		Reverse	CACAGCATAACACACATCGAGAA

## Abbreviations

DEGs: differentially expressed genes
EAC: esophageal adenocarcinoma
ESCC: Esophageal squamous cell carcinoma
GEO: Gene Expression Omnibus
GO: Gene ontology
MCC: Maximal Clique Centrality
NCBI: National Center for Biotechnology Information
PCA: principal component analysis
PPI: protein‑protein interaction
qPCR: real-time quantitative PCR
scRNA-seq: single cell RNA sequencing
TCGA: The Cancer Genome Atlas
ROC: receiver operating curve
TIMER: Tumor Immune Estimation Resource
